# Quantification of Phosphorylated Tau Protein in Alzheimer's Disease Patients’ Fluids With Electrolyte‐Gated Organic Transistor Biosensors

**DOI:** 10.1002/adhm.71634

**Published:** 2026-08-27

**Authors:** Marcello Berto, Pamela A. Manco Urbina, Alessandro Paradisi, Matteo Sensi, Najara Iacovino, Chiara Carbone, Roberta Bedin, Marcello Pinti, Fabio Biscarini, Giovanna Zamboni, Carlo A. Bortolotti

**Affiliations:** ^1^ Dipartimento di Scienze Della Vita Università di Modena and Reggio Emilia Modena Italy; ^2^ Dipartimento di Scienze Biomediche, Metaboliche e Neuroscienze Università di Modena and Reggio Emilia Modena Italy; ^3^ Center For Translational Neurophysiology of Speech and Communication Istituto Italiano di Tecnologia Ferrara Italy; ^4^ Azienda Ospedaliero Universitaria di Modena Modena Italy

**Keywords:** affinity constant, cerebrospinal fluid, dementia, EGOT, gray matter density, phosphorylated tau protein 181, voxel‐based morphometry

## Abstract

Dementia is a syndrome that affects millions of people in the world, and Alzheimer's disease (AD) is the most common cause. The diagnosis of AD begins with cognitive symptoms such as mild cognitive impairment (MCI) but requires the demonstration and quantification of the underlying neuropathological processes through the measurement of specific biomarkers. Among these, the phosphorylated tau protein at threonine 181 (p‐tau 181) is considered specific for AD diagnosis in people with MCI. In this work, we report the detection of p‐tau 181 spanning from physiological to pathological concentrations in cerebrospinal fluids from patients with MCI. The proposed sensor is based on an electrolyte‐gated organic transistor functionalized with specific anti‐p‐tau 181 antibodies. We analyzed the multiparametric sensor response using a Langmuir model to extract the thermodynamic affinity constant, resulting in values between 10^12^ and 10^13^. The sensor response is then correlated with the gray matter volume of the same patients from Magnetic Resonance Imaging: the resulting maps show areas in the brain where the gray matter density decreases with increasing levels of p‐tau in CSF.

## Graphical abstract

Dementia syndrome affects millions of people in the world, and Alzheimer's disease (AD) is the most common cause. We report the detection of the specific biomarker for AD, p‐tau‐181, from physiological to pathological concentrations in cerebrospinal fluid using an electrolyte‐gated organic transistor biosensor. The response is correlated with the gray matter volume loss of the patients from Magnetic Resonance Imaging.

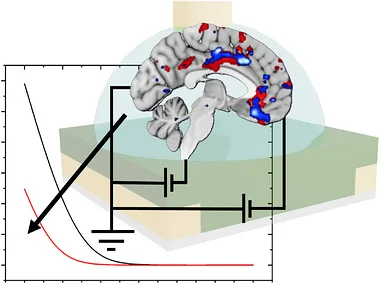

## 1 Introduction

Alzheimer's disease (AD) is the most common cause of dementia worldwide [1]. Nowadays more than 50 million people in the world are living with AD or other dementias and this number can triple by 2050 as the result of global population aging [2, 3]. The socio‐economic cost consisting in direct medical costs and indirect costs is considerable [4], and it is estimated as high as $2.11 trillion in 2030, based on the World Alzheimer Report [2, 5, 6].

AD is a biological process that gives rise to a spectrum of clinical manifestations, ranging from very subtle or preclinical cognitive symptoms to mild cognitive impairment (MCI) and, ultimately, to overt dementia. Mild cognitive impairment (MCI) represents an intermediate clinical stage, defined by the presence of objectively measurable cognitive symptoms, such as memory loss, yet not affecting functional independence in activities of daily living [7, 8]. The diagnosis of AD requires the demonstration and quantification of the underlying neuropathological processes through the measurement of specific biomarkers. Nowadays, these biomarkers are measured with imaging techniques such as positron emission tomography (PET) or quantified in the cerebrospinal fluid (CSF) collected by lumbar puncture [7, 9, 10, 11, 12]. Among these biomarkers, the pathological increase of the phosphorylated tau protein at threonine 181 (p‐tau 181) in the CSF is considered specific for AD diagnosis in people with MCI, since it is correlated both with the phosphorylation state of tau and the formation of neurofibrillary tangles in the brain [13, 14, 15, 16]. Although CSF sampling is more invasive than blood‐based testing, CSF biomarkers are routinely used in specialized memory clinics, including our center. Thus, CSF‐based validation provides comparison with an established clinical biomarker reference standard.

**FIGURE 1:**
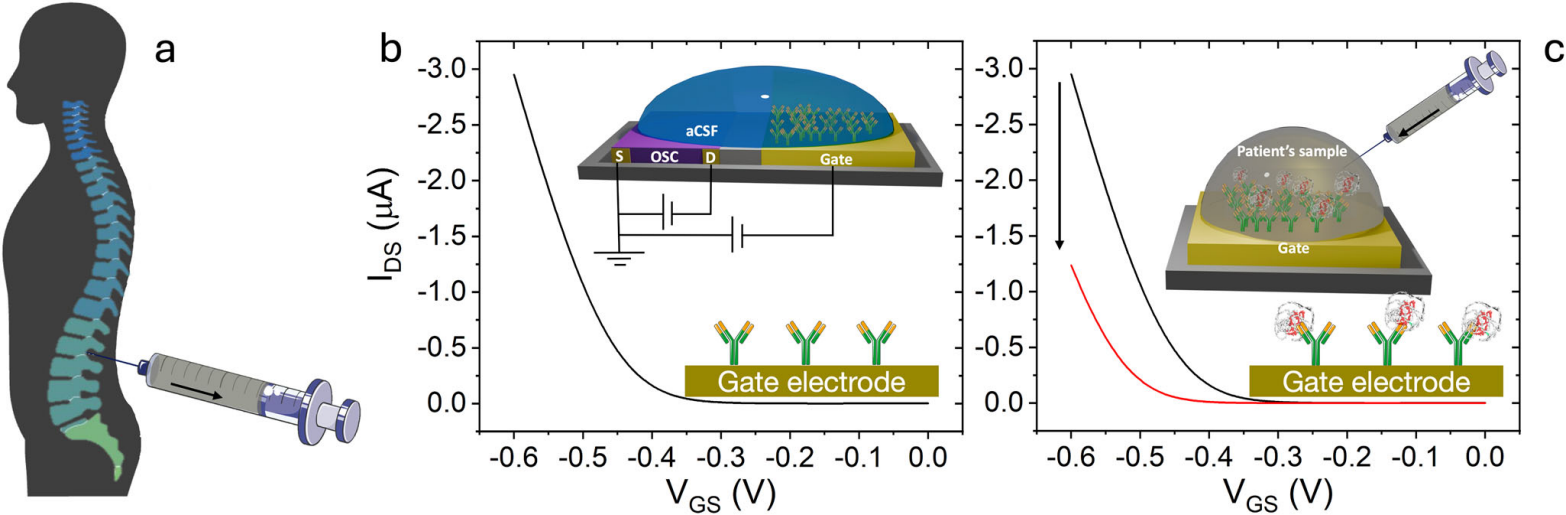
(a) Collection of CSF samples by lumbar puncture. (b) Schematic of the planar gate organic transistor used in this work: S and D are source and drain electrodes, respectively, aCSF is artificial cerebrospinal fluid, and OSC is the organic semiconductor. (c) Change of the transfer curve of the EGOT sensor following incubation of the sensing gate in the patient's CSF.

In this work, we demonstrate the quantification of p‐tau 181 in CSF with ad hoc designed label‐free biosensors that specifically recognize this protein. Samples were obtained by lumbar puncture from ten patients with MCI (Figure 1a), in a window of p‐tau 181 concentrations spanning a range from physiological (≤56.5 pg/mL) to pathological (≥56.5 pg/mL) levels [9, 12, 17]. Among other analytical techniques, mass spectrometry (MS) has been demonstrated for the quantification of phosphorylated tau protein levels in bodily fluids. Nevertheless, the need for pre‐treatment steps of the samples before the analysis (e.g., MS coupled to liquid chromatography, immunoprecipitation of the tau isoforms) [18, 19, 20] can be a strong limitation in the use of this technique. The use of organic electronic biosensors, which are nowadays state‐of‐the‐art sensing devices, opens unprecedented possibilities, ensuring high sensitivity and rapid label‐free response at the point of care (PoC), while keeping the costs of the disposable sensors low and without the need for pre‐treatment steps [21, 22, 23, 24].

We built the dose curve in CSF for the p‐tau EGOT sensor following validated clinical quantification of this biomarker with Lumipulse chemiluminescent assay [10].

Furthermore, we correlate the resulting p‐tau values from both the EGOT sensor and the chemiluminescent assay with the outcome from patterns of brain volume loss (i.e., brain atrophy) using voxel‐based morphometry (VBM), a well‐established imaging technique which allows the detection of associations between interindividual structural variability in regional brain atrophy and specific clinical variables [25, 26].

Electrolyte‐gated organic transistors (EGOTs) are convenient architectures for biosensing as they can be made specific to the relevant target by the chemical functionalization of the gate electrode/electrolyte interface [27, 28, 29, 30, 31, 32, 33], typically by immobilizing an antibody (Ab) at the gate surface [34, 35, 36]. In the last years, EGOT‐based biosensors have been proposed for the detection of analytes on different length scales, from small molecules [31, 37], to proteins [35, 38, 39], to DNA [40, 41], and even to large viruses [42] or lipid vesicles [43, 44]. Figure 1b shows a schematic drawing of the EGOT biosensor in a planar side gate layout. In this study, the electrolyte bridging gate and channel (made of an organic semiconductor thin film) is artificial CSF (aCSF), whose composition is reported in Table S1. The high sensitivity of EGOT biosensors derives from their ability to amplify small variations of interfacial electrostatic potentials into large detectable current signals by means of the EGOT large effective capacitance [28, 45, 46]. This desirable feature could negatively affect the sensor response in the presence of spurious signals due to nonspecific binding events at one relevant interface. As a consequence, biosensing in biological fluids can be jeopardized by the presence of a number of potential nonspecific targets [47], thus making differential detection with respect to control samples fundamental. To limit nonspecific signals, the measurement is carried out with a pristine functionalized gate electrode first incubated into aCSF, then with the same gate electrode incubated ex situ in the patient sample and measured in aCSF as depicted in Figure 1c.

## 2 Results and Discussion

**FIGURE 2:**
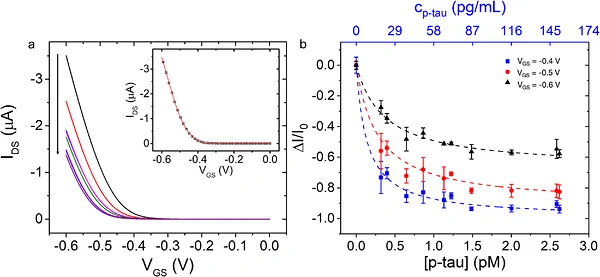
(a) Transfer characteristics of the EGOT sensor at increasing c_p‐tau_ from 0 to 152 pg/mL. Inset: representative transfer curve (squares) fitted with eq. 18 from ref. [30] (continuous red lines). (b) Dose curves acquired at three different gate voltage values, that is, *V*_GS_ = −0.6 V (black triangles), *V*_GS_  = −0.5 V (red circles), and *V*_GS_  = −0.4 V (blue squares). Dashed lines are the best fit with the Langmuir model (Equation 1). The error bars correspond to the standard deviation of four independent experiments.

To realize the EGOT‐based biosensor, we functionalized the gold gate electrode with a specific anti‐p‐tau 181 antibody. The correct and uniform orientation of the antibodies, necessary to optimize the sensing response of the EGOT biosensor [48], was achieved through the immobilization of the antibodies to a sub‐monolayer of the recombinant protein G deposited on the gold substrate by a cysteine. Protein G has high affinity for the heavy chain of Immunoglobulin G, thus it grafts antibodies on the gate surface with a precise orientation. After protein G and antibodies immobilization, we deposited a (11‐mercaptoundecyl)tri(ethylene glycol) (OEG) self‐assembled monolayer to minimize nonspecific adsorption to the gold surface not covered by the protein G/antibodies [42]. We checked the efficacy of the protein G coverage through cyclic voltammetry in presence of the redox probe Fe(CN)_6_^3–/4−^ connecting the gate as the working electrode in a standard three‐electrodes configuration (results reported in Figure S1). From the electrochemical characterization using the Randles–Ševčík equation [49] we estimate the coverage of the gate surface by protein G to be 30% ( ± 9%). The ability of the EGOT sensor to detect and quantify p‐tau in cerebrospinal fluid was demonstrated by incubating the functionalized gate electrode in patients’ samples containing increasing concentrations of p‐tau (previously assessed by chemiluminescent assay), then acquiring the transfer characteristics in aCSF. Figure 2a shows the overlay of transfer curves after exposure to increasing concentration of p‐tau: a shift of the curves to more negative *V_GS_* values is observed, that is, the transistor turns on at more negative *V_GS_* values with increasing concentration of p‐tau, causing a decrease of the current *I_DS_* at any gate voltage where the EGOT is on. To build a quantitative dose curve from the EGOT response, we plotted the relative variation of *I_DS_* at different *V_GS_* for every [p‐tau] with respect to the *I_0_* value in absence of the target. The resulting dose curves are reported in Figure 2b. We observe an amplification of the response at lower *V_GS_*, with the largest current variations obtained when EGOT is operated at *V_GS_* = −0.4 V, namely, in the subthreshold regime [38, 50] (blue squares in Figure 2b). The trend of the data hints to a saturation plateau at the highest concentrations. We fit the dose curves with a Langmuir function (Equation 1) to extract the thermodynamic affinity constant *K_a_*.

(1) $$\;\frac{\Delta I}{I_{0}}={\left(\frac{\Delta I}{I_{0}}\right)}_{max}\;\cdot \frac{K_{a}\cdot \left[p-tau\right]}{1+K_{a}\cdot \left[p-tau\right]}$$

where *ΔI/I_0_* is the relative variation of the current between source and drain *I_DS_* at every molar concentration [p‐tau] and the current *I_0_* at [p‐tau] = 0 M, both extracted at the same *V_GS_* [51]; (*ΔI/I_0_*)*_max_* is the maximum variation of current *I_DS_*; *K_a_* is the affinity constant. The MW of p‐tau is taken as 58 kDa [52, 53, 54].

By fitting the data reported in Figure 2b, we obtained the *K_a_* values for the binding process between p‐tau 181 in solution and the specific antibody immobilized on the gate surface, ranging from (7.1 ± 2)·10^12^ at *V_GS_* = −0.4 V, to (2.7 ± 0.4)·10^12^ at *V_GS_* = −0.6 V. The apparent dependence of the *K_a_* value on the gate potential *V_GS_* has been discussed in previous publications [38, 39, 50]. Here we only comment on the fact that the large *K_a_* values (10^12^–10^13^) indicate that our EGOT sensor is on the one hand highly specific toward p‐tau; on the other hand, the most sensitive operation mode (viz. in sub‐threshold regime) will attain the saturation of the response (approximately at concentration above 2/*K_a_*) at lower concentration, and hence will be insensitive to the pathological levels. This opens the consideration that the operation mode of the EGOT sensor should be investigated at different gate potentials for quantification of p‐tau in the pathological range, which can be achieved at the cost of a decreased sensitivity at the lowest concentration in favor of yielding a variability of the signal at the concentrations >56.5 pg/mL.

**FIGURE 3:**
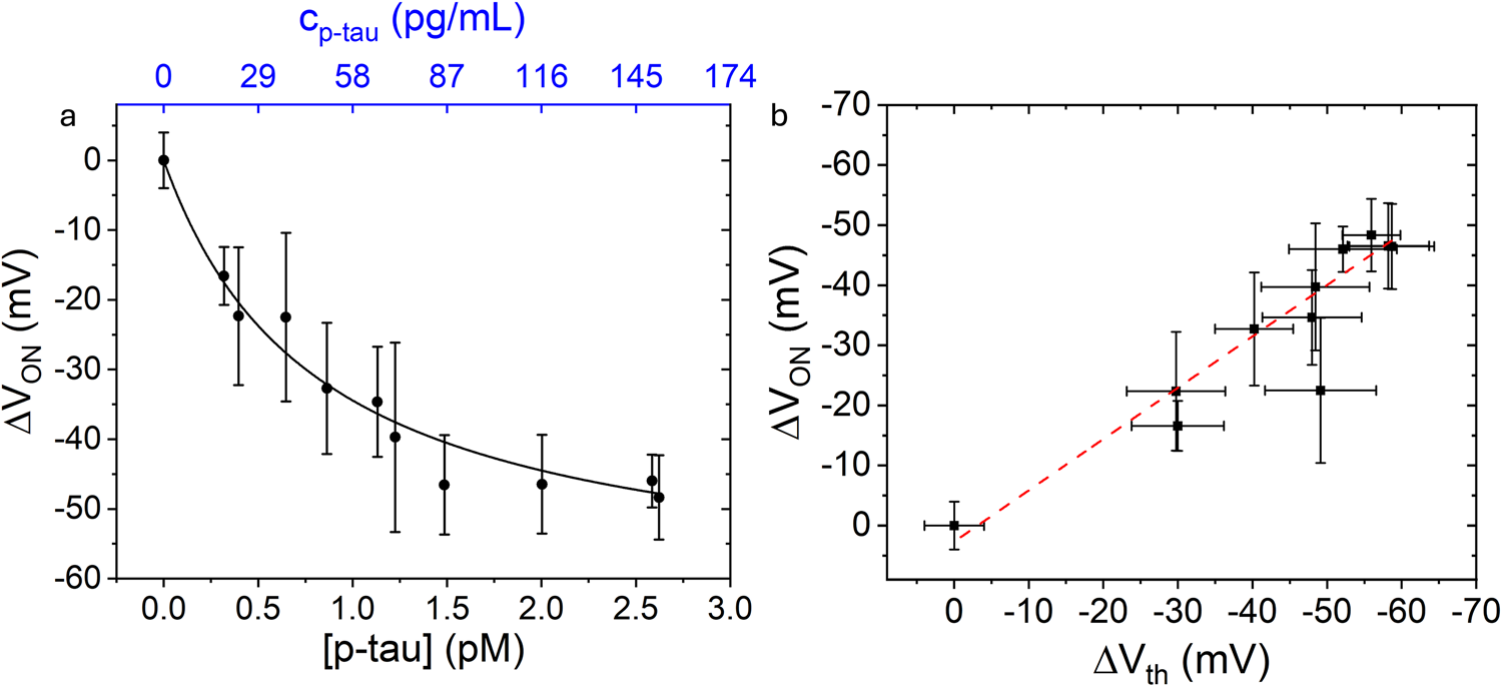
(a) Switch‐on voltage shift as a function of p‐tau concentration. The black line is the best fit with the Langmuir model (Equation 1). (b) Correlation plot between Δ*V*_th_ (horizontal axis) and Δ*V*_ON_ (vertical axis). The dashed red line slope is 0.83. The error bars correspond to the standard deviation of four independent experiments.

It has been demonstrated that the biorecognition events at the gate‐electrolyte interface affect the electrical response of the transistor sensors [27, 30, 34, 38, 55]. In this work, we adopt the model proposed by Zanotti [30] to analyze these correlations: The model describes how the density of charge carriers in the organic semiconductor (positive charge carriers in the proposed p‐type semiconductor DPP‐DTT) influences the effective capacitance of the transistor architecture, hence its electrical behavior. In the present case, the biorecognition events at the gate‐electrolyte interface result in a variation of the output current of the device, as reported in Figure 2a. To deepen the analysis of the EGOT sensor behavior, we fitted the transfer curves with eq. 18 reported in reference [30] (a representative result is reported in the inset of Figure 2a). We used it to extract additional parameters whose variation is correlated to [p‐tau]. One of these parameters is the switch‐on voltage *V_ON_*, defined as the gate voltage value needed to maintain the organic semiconductor in a charge neutrality condition (flat band potential) [30]. Variations of the work function of the gate electrode due to biorecognition events cause a shift of the *V_ON_* proportional to the amount of analyte in solution. In Figure 3a, we report the variation of *V_ON_* as a function of [p‐tau]: *V_ON_* shifts monotonically toward more negative values down to ∼−50 mV at the highest p‐tau concentration tested, namely 152 pg/mL. We then compared the variation of *V_ON_* with the variation of the threshold voltage *V_th_*, defined as the gate voltage where a conducting channel forms between the source and the drain electrodes [56].

*V_th_* values can be calculated using Equation (2) from the transfer curves acquired at increasing p‐tau concentrations (Figure 2a):

(2) $$\;I_{DS}=\frac{W}{L}\;\mu_{\left(h\right)}C\left(V_{GS}-V_{th}\right)V_{DS}$$

where *W/L* is a geometric parameter describing the ratio between the width (*W*) and the length (*L*) of the active channel, *μ_(h)_* is the charge carrier mobility (holes) in the channel, and *C* is the effective capacitance of the EGOT coupling the gate with the channel. The panel in Figure S2a reporting *ΔV_th_* and *ΔV_ON_* versus [p‐tau] and the correlation plot in Figure 3b both show that the trends of *V_ON_* and *V_th_* are similar, but the variation of *V_ON_* is smaller than that of *V_th_*. In particular, *ΔV_ON_* is approximately 80% of *ΔV_th_*, reaching a maximum variation of ∼−50 mV, while the maximum *ΔV_th_* ∼−60 mV. We fit both *ΔV_on_‐* and *ΔV_th_‐*based dose curves with a Langmuir model (Equation 1), extracting an affinity constant of (2.7 ± 0.8)·10^12^ for *ΔV_th_* and (1.2 ± 0.2)·10^12^ for *ΔV_ON_*.

Other parameters that can be extracted from the fitting of the transfer curves are the linear transconductance *g_m_*, defined as the amplification capability of the transistor, and the energy disorder of the organic semiconductor *σ*. The change of the effective potential at the gate electrode due to the biorecognition events impacts on how the ions contained in the electrolyte solution penetrate the DPP‐DTT, thus modifying the energy disorder *σ* of the organic semiconductor film. As shown in Figure S2 all these parameters can be used to build the corresponding dose curves. It is evident that they all contribute to the relative variation of the current reported in Figure 2, but the errors associated with *V_th_* and *V_ON_* values are smaller than the ones on *g_m_* or *σ*. The sensing mechanism upon the recognition process relies on the variation of the electrochemical potential of the gate electrode, while the active channel is involved mainly in the transduction operation. Thus, the parameters more sensitive to the biorecognition events are the ones influenced by the variation of the gate potential, namely, *V_th_* and *I_DS_*. Using the Langmuir model (Equation 1), we calculated the affinity constant *K_a_* values for the couple p‐tau 181/Antibody for all the parameters extracted. The results are summarized in Table S2: calculated *K_a_* values based on *ΔI/I_0_* at *V_GS_* = −0.6 V, *ΔV_th_*, *ΔV_ON_, Δ g_m_/g_m,0_* and *Δσ* all lie between 1.2·10^12^ and 2.7·10^12^, thus demonstrating the robustness of the analysis. Conversely, in line with previous results from our group, the *K_a_* values extracted from *ΔI/I_0_* is affected by the gate potentials at which the current difference is measured, and this leads to variation in *K_a_* up to about one order of magnitude [38, 39].

We demonstrated with control experiments that the variation of the sensor response is related to the specific recognition of p‐tau by the corresponding antibody immobilized on the gate surface. CSF is a complex matrix containing a number of ions and biomolecules, such as vitamins, peptides, growth factors, and proteins [57, 58, 59], thus leading to potentially nonspecific signals after incubation of the sensing electrode. Since p‐tau protein is always present at different levels in the CSF samples, we functionalized the sensing gate electrode with a nonspecific antibody immobilized on the protein G sub‐monolayer, and we performed the sensing experiments at increasing p‐tau concentrations, recording transfer curves after the incubation of the gate electrode in the patient's CSF. The magnitude of the nonspecific response at the highest [p‐tau] tested, namely 116 pg/mL, is more than five times lower than the specific one (Figure S3). Moreover, we do not observe a monotonical variation of the output current with p‐tau concentration over the investigated range (Figure S4), thus supporting the hypothesis that the device response is proportional to the amount of p‐tau in solution.

In order to validate the label‐free detection and quantification of p‐tau in CSF, we compared the current signal (at *V_GS_* = −0.6 V) from the EGOT sensor with the results obtained by the Lumipulse chemiluminescent assay [10]. The latter assay is an established and validated diagnostic technique for biomarkers of AD, used in routine clinical practice at the Modena University Hospital and internationally. Therefore, it could be considered a reference standard or benchmark method for assessing the performance of our device. Established cutoff values for CSF p‐tau concentration measured with a chemiluminescent assay is 56.5 pg/mL.

Pearson correlation analyses revealed a strong positive association between CSF p‐tau concentration obtained with chemiluminescent assay and the EGOT signal calculated at all three *V_GS_* reported (*r* = 0.81–0.85, all *p* < 0.05). Formal comparison using Fisher r‐to‐z transformation showed no significant differences between correlation coefficients (all *p* > 0.80), indicating a stable and robust relationship between chemiluminescence values and the EGOT measurements_._

**FIGURE 4:**
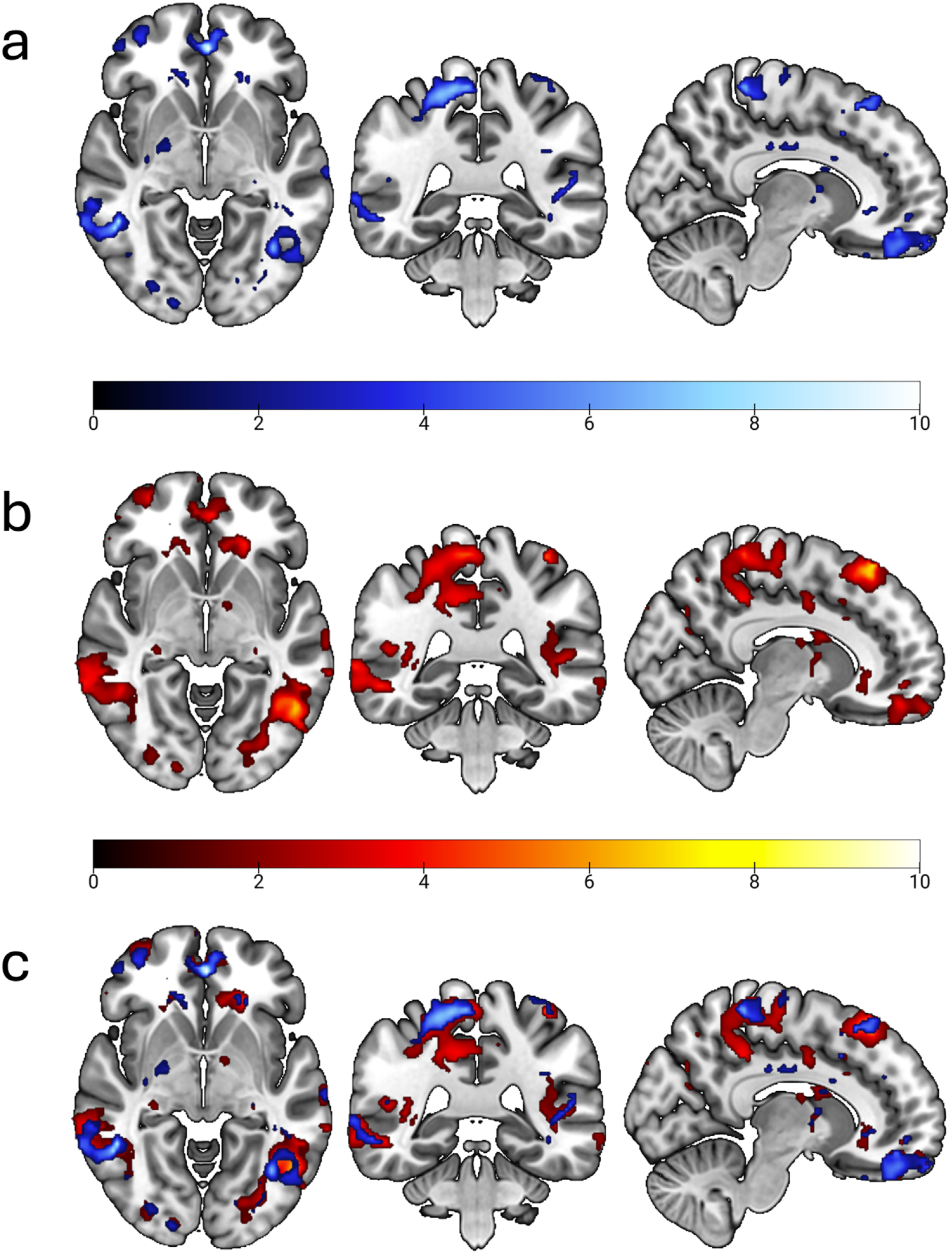
(a) Regions of significant VBM correlation between gray matter volume and p‐tau measured with the EGOT sensor at *V_GS_* = −0.6 V (*p*<0.005, uncorrected). The *t*‐value of the Student's *t*‐test is displayed as blue scale. (b) Regions of significant VBM correlation between gray matter volume and p‐tau measured with the Lumipulse chemiluminescent assay. The *t*‐value of the Student's *t*‐test is displayed as red scale. (c) Overlay between (a) and (b). The anatomical regions visualized in gray scale are not correlated or correlation is below the threshold value.

To further explore the potential clinical relevance of the measurements of CSF p‐tau obtained with the EGOT sensor, we correlated the EGOT current signal to measurements of regional structural brain damage (i.e., gray matter loss, also termed brain atrophy). Brain atrophy was measured on high‐resolution MRI imaging acquired at the 3T scanner of the Modena University Hospital on the same ten MCI patients. MRI data were acquired in close temporal proximity (<1 month) to CSF samples. Correlational VBM allows the identification of brain regions (groups of voxels, the unit of volume of 1 mm^3^) in which the density of gray matter linearly correlates with a variable of interest (in our case, the current signal measured by the EGOT sensor) across the group of ten patients with MCI [25, 26]. Correlational VBM gives as a result a brain map representing the regions in which the brain atrophy is greater with increasing values of CSF p‐tau; hence, it displays the brain regions where patients with high CSF p‐tau levels exhibit greater damage by the neurodegenerative process. The results are reported in Figure 4 as color maps overlapped on projections of a standard template brain. The blue color bar reports the *t*‐value of the Student's *t*‐test. The anatomical regions visualized in gray scale are correlated below the statistical threshold of *p* < 0.005, uncorrected for multiple comparisons. We found that, at this statistical threshold, the patients showing the highest CSF p‐tau values according to the current signal of the EGOT sensor at *V_GS_* = −0.6 V also exhibit the lowest gray matter density in regions of the medial prefrontal cortex and the lateral parietal regions (Figure 4a). These are associative brain areas not primarily involved in atrophy in AD but known to become hypometabolic in neurodegenerative diseases including AD. Another correlational VBM was performed using the CSF p‐tau measured with the benchmark test based on a chemiluminescent assay (Figure 4b). The red color bar reports the *t*‐value of the Student's *t*‐test. The overlay of the two resulting maps in Figure 4a,b is shown in Figure 4c. There are important brain regions where the blue and the red maps overlap, which supports the significance of the EGOT sensor measurements. Yet we observe that the correlation with the chemiluminescent assay evidences additional areas which may be ascribed to the fact that the chemiluminescent assay accesses a larger dynamic range. Correlation analysis between gray matter volume and EGOT current signals at *V_GS_* = −0.4 V and *V_GS_* = −0.5 V also gave similar brain maps (not shown).

## 3 Conclusion

We present a specific sensor based on EGOT devices for the quantification of the phosphorylated tau protein at threonine 181 (p‐tau 181) in cerebrospinal fluid. In patients with MCI, the presence of pathological levels of p‐tau 181 is among the biomarkers supporting the diagnosis of AD as the cause of the cognitive symptoms, together with the presence of atrophy of specific areas of the brain. Our EGOT sensor exploits the biorecognition between p‐tau in solution and the specific antibody immobilized on the gate electrode surface to quantify the protein levels in CSF samples from MCI patients. Notwithstanding the complexity of the patients’ samples, the EGOT sensor shows a specific multiparametric response, spanning from the physiological to the pathological levels of p‐tau. We analyzed the sensor response using a Langmuir model to extract the thermodynamic affinity constant of our sensing element, resulting in very high values between 10^12^ and 10^13^. To explore the ability of the EGOT sensor to provide information complementing the outcome of routine clinical practices, we correlate the levels of p‐tau obtained with the EGOT sensor with the gray matter volume of the same patients: the resulting maps show areas in the brain where the gray matter density decreases with increasing levels of p‐tau in CSF.

We believe that this work shows the potentiality of EGOT‐based biosensors for the quantification of AD‐specific biomarkers, both in the early stages of the disease and in pathological conditions. In the future, we can envision the implementation of the EGOT sensor for the quantification of p‐tau in CSF as an additional tool in the AD diagnostic routine together with neurologic diagnosis of MCI and MRI analysis of the brain.

## 4 Materials and Methods

### 4.1 Materials

All the reagents and materials were used as provided by the manufacturer without further purification. The poly[2,5‐(2‐octyldodecyl)‐3,6‐diketopyrrolopyrrole‐alt‐5,5‐(2,5‐di(thien‐2‐yl)thieno [3,2‐b]thiophene)] (DPP‐DTT) was purchased from Ossila Ltd. 11‐Mercaptoundecyl)tri(ethylene glycol) (OEG) was purchased from Merck. Cys‐protein G was purchased from Bio‐Techne. Anti‐p tau 181 antibody was purchased from Life Technologies Italia.

### 4.2 Device Fabrication

The EGOT transistors were fabricated using gold interdigitated source and drain electrodes patterned on glass substrates by Fondazione Bruno Kessler FBK, Trento (Italy). Prior to depositing the organic semiconductor, the substrates were cleaned according to the following procedure: i) rinsing with acetone, ii) drying gently with nitrogen flow, iii) etching in piranha solution (1:1 ratio of H_2_SO_4_:H_2_O_2_) at 150°C for 5 min, and iv) a final rinsing with water. After the cleaning procedure, the substrates were dried gently under nitrogen flow. The semiconductor DPP‐DTT was deposited on the substrates by spin coating from a 5 mg/mL solution in 1,2‐chlorobenzene and then cured in a thermostatic oven at 140°C for 30 min.

### 4.3 Gate Functionalization

The gate electrodes are fabricated on Kapton substrates by PoliFAB (Milano, Italy). The gold gate electrode were functionalized according to the following immobilization protocol: (i) a first incubation in a phosphate buffer saline (PBS 50 mM, pH 7.4) of Cys‐Protein G (2 mg/mL) overnight at 4°C, (ii) rinsing with PBS, (iii) incubation with an anti‐p tau 181 (0.1 mg/mL) solution for 1 h at RT (iv) rinsing with PBS, (v) immersion in OEG thiol 100 µM (in PBS 50 mM, pH 7.4) for 20 min and (vi) a final rinse with PBS.

We checked the protein G coverage and all the functionalization steps by electrochemical techniques (cyclic voltammetry and electrochemical impedance spectroscopy). The measurements were performed in 5 mM K_3_[Fe(CN)_6_], 1 M KCl at 50 mV s^−1^. The experiments were carried out in a three‐electrode cell at room temperature using a CH Instrument potentiostat 760c model: The gold gate electrode was used as the working electrode (WE), a platinum wire was used as the counter electrode (CE), and an Ag/AgCl electrode was used as the reference electrode (RE). The results reported in Figure S1 show the effect on electron transfer and interfacial capacitance of the WE after every step.

### 4.4 Electrical Characterization

All the electrical measurements were performed at room temperature inside a Faraday cage using an Agilent B2902A Source Measure Unit.

A planar rectangular [8 × 4 mm^2^] gold gate electrode patterned on Kapton substrate was used as the gate electrode. Transfer characteristics were acquired by sweeping gate‐source potential (*V_GS_*) between 0 and −0.6 V keeping *V_DS_* = −0.1 V. Prior to performing the sensing experiments, the devices were cycled to stability using artificial cerebrospinal fluid (aCSF) as electrolytic solution. The composition of aCSF is reported in Table S1. Before electrical measurement the gate electrodes were incubated ex situ in CSF samples from patients containing increasing [p‐tau]. The reproducibility of the fabricated EGOTs is good, as demonstrated by the average values of two important figures of merit, viz. the transconductance *g_m_* = (13.5 ± 2) µS and the threshold voltage *V_th_* = (−0.44 ± 0.02) V.

### 4.5 Clinical Population

Patients with a clinical diagnosis of Mild Cognitive Impairment (MCI) [60] from the Cognitive Neurology Clinic of Modena University Hospital, Northern Italy, gave written informed consent for the use of their cerebrospinal fluid (CSF) samples and imaging data for the purpose of the present study, which had had been approved by the local Ethics Committee (Comitato Etico dell'Area Vasta Emilia Nord, approval no 156/2022/TESS/AOUMO). All subjects had undergone neurological examination, extensive neuropsychological assessment, blood exam, CSF collection, and magnetic resonance imaging (MRI). CSF collection and sample handling followed Standard International Procedures for biobanking [PMID 26821803]. Upon arrival at the neuro‐immunology laboratory within the same hospital, samples were anonymized using alphanumeric codes and processed within 30 min. After centrifugation at 2500×g for 10 min at controlled room temperature, the supernatants were aliquoted into sterile polypropylene vials (1.5 mL Microtube, Sarstedt AG&Co) and stored at −80°C until analysis. Prior to testing, samples were thawed and re‐centrifuged at 2500×g for 5 min according to the manufacturer's instructions.

### 4.6 Lumipulse Assay

The Lumipulse chemiluminescent assay was used as reference standard. Analysis was conducted using the Lumipulse G600II fully automated system with Lumipulse G p‐Tau181 Chemiluminescent Enzyme ImmunoAssays (Fujirebio Inc., Ghent, Belgium). A p‐Tau181 concentration of <56.5 pg/mL was considered below the laboratory‐specific cut‐off. Analyses were performed by biologists blinded to the participants’ clinical data.

### 4.7 MRI Acquisition, Processing and Analyses

MRI data were acquired on a 3T GE Signa Architect scanner in Modena University Hospital equipped with a 48‐channel‐array head coil. The multimodal MRI protocol included, among other sequences, high‐resolution T1‐weighted 3D MP‐RAGE structural images (TR 2 sec; TE 3.1 msec; FOV 172 × 256 × 256 mm^3^; voxel dimension 1 mm isotropic). Images were analyzed using FSL (FMRIB Software Library) v6.0 software (https://fsl.fmrib.ox.ac.uk/fsl) [26]. The ten structural images were first brain‐extracted, gray matter‐segmented, and registered to the Montreal Neurological Institute's 152 standard space using nonlinear registration at the single‐subject level [61]. Then, a study‐specific gray matter template was created, and all structural images were nonlinearly registered to that, modulated, and finally smoothed with an isotropic Gaussian kernel with a sigma of 3 mm. The relationship between gray matter volume and p‐tau was examined using one‐tailed *t‐*tests, assuming that increasing levels of p‐tau would be associated with decreased tissue density/volume. Whole brain data were analyzed setting the voxel‐level statistical threshold at *p*<0.005, uncorrected, given the exploratory nature of the present study and the small sample size (ten MCI patients). To identify regions of significant correlation between gray matter density and p‐tau, we used permutation‐based nonparametric inference within the framework of the general linear model (5000 permutations) [25]. Fisher r‐to‐z transformation was used exclusively for the comparison of subject‐level correlation coefficients between EGOT and Lumipulse measurements. Conversely, VBM analyses were performed voxel‐wise in FSL using the general linear model, in which the association between gray matter volume and p‐tau‐related measures is tested independently at each voxel and expressed as a *t*‐statistic.

### 4.8 Statistical Analysis

To present the electronic data, we used the mean of the last five transfer curves registered after stabilization to build the dose curves. The variation of the output current is normalized with respect to the value registered in absence of the analyte. Data are presented as the mean ± standard deviation (SD) with the number of independent replicates specified in the figure captions. All the electronic data have been analyzed and plotted using OriginPro.

CSF samples were processed according to standardized biobanking procedures, and p‐tau181 concentrations were quantified using the Lumipulse assay as reference standard. No transformation or normalization was applied to these variables before correlation analyses. MRI data were pre‐processed in FSL with brain extraction, gray matter segmentation, nonlinear registration to standard space, creation of a study‐specific gray matter template, modulation, and spatial smoothing with an isotropic Gaussian kernel with sigma = 3 mm. Data quality was assessed during experimental acquisition and image pre‐processing; no subject was excluded as an outlier.

Data are presented as mean ± standard deviation (SD) unless otherwise specified. Clinical, biochemical, and MRI correlation analyses were performed in 10 patients with MCI. Pearson correlation analyses were used to assess the association between CSF p‐tau181 concentrations measured by Lumipulse and EGOT‐derived current signals at the three tested gate voltages. Statistical significance was assessed using two‐sided tests with an alpha level of 0.05. Fisher r‐to‐z transformation was used exclusively to compare subject‐level correlation coefficients between EGOT and Lumipulse measurements. No post‐hoc correction was applied to these comparisons, given their descriptive validation purpose and the small exploratory dataset.

VBM analyses were performed in FSL using the general linear model. The relationship between gray matter volume and p‐tau‐related measures was tested independently at each voxel using one‐tailed *t*‐tests, based on the a priori hypothesis that higher p‐tau levels would be associated with lower gray matter density/volume. Statistical inference was performed using permutation‐based nonparametric testing with 5000 permutations. Whole‐brain results were displayed at a voxel‐level threshold of *p* < 0.005, uncorrected for multiple comparisons, given the exploratory nature of the study and the limited sample size. VBM results are reported as t‐statistic maps. MRI pre‐processing and voxel‐wise statistical analyses were performed using FSL v6.0.

## Author Contributions

**Marcello Berto**: conceptualization, methodology, investigation, visualization, Writing – original draft. **Pamela A. Manco Urbina**: investigation, visualization. **Alessandro Paradisi**: conceptualization, investigation, visualization, writing – review & editing. **Matteo Sensi**: visualization, writing – review & editing. **Najara Iacovino**: formal analysis, investigation, writing – review & editing. **Chiara Carbone**: formal analysis, investigation, writing – review & editing. **Roberta Bedin**: formal analysis, investigation, writing – review & editing. **Marcello Pinti**: resources, writing – review & editing. **Fabio Biscarini**: supervision, resources, writing – review & editing. **Giovanna Zamboni**: conceptualization, supervision, resources, funding acquisition, writing – review & editing. **Carlo A. Bortolotti**: conceptualization, supervision, resources, funding acquisition, writing – review & editing.

## Funding

The research leading to these results has received funding from the European Union—NextGenerationEU through the Italian Ministry of University and Research under PNRR—M4C2‐I1.3 Project PE_00000019 “HEAL ITALIA” (CUP E93C22001860006) to Carlo Augusto Bortolotti and Marcello Berto. Authors acknowledge funding from the University of Modena and Reggio Emilia through ‘FAR 2021’ Fondazione di Modena. This research was funded under the project “[FIS‐2024‐05091 HELP Hierarchical Organic Electronics Sensors for Phenotyping Alzheimer's and Inflammatory Diseases at the Point of Care]”, CUP [E53C25002440001], supported by the Fondo Italiano per la Scienza—FIS 3. Financial contribution from the PRD2026 from the Department of Life Sciences, University of Modena and Reggio Emilia, Italy, is gratefully acknowledged. The views and opinions expressed are those of the authors only and do not necessarily reflect those of the European Union or the European Commission. Neither the European Union nor the European Commission can be held responsible for them.

## Conflicts of Interest

The authors declare no conflicts of interest.

## Supporting information




**Supporting File**: adhm71634‐sup‐0001‐SuppMat.docx.

## Data Availability

The data that support the findings of this study are available from the corresponding author upon reasonable request.
