# Supplementary material for: Quantification of Phosphorylated Tau Protein in Alzheimer's Disease Patients’ Fluids With Electrolyte‐Gated Organic Transistor Biosensors
Source: Adv Healthc Mater. 2026 Aug 27;15(36):e71634. doi: 10.1002/adhm.71634 (PMC13612928; doi:10.1002/adhm.71634)
Supplement: Supplementary file 1 [file ADHM-15-0-s001.docx]

**SUPPORTING INFORMATION**

**Quantification of phosphorylated tau protein in Alzheimer’s Disease patients’ fluids with electrolyte-gated organic transistor biosensors**

Marcello Berto^a^, Pamela A. Manco Urbina^a^, Alessandro Paradisi^a^, Matteo Sensi^a^, Najara Iacovino^b^, Chiara Carbone^b^, Roberta Bedin^b^, Marcello Pinti^a^, Fabio Biscarini^a,c^, Giovanna Zamboni^b,d*^, Carlo A. Bortolotti^a*^

a) Dipartimento di Scienze della Vita, Università di Modena and Reggio Emilia, Via Giuseppe Campi 103, Modena, 41125 Italy
b) Dipartimento di Scienze Biomediche, Metaboliche e Neuroscienze, Università di Modena and Reggio Emilia, Via Giuseppe Campi 287, Modena, 41125 Italy
c) Center for Translational Neurophysiology of Speech and Communication, Istituto Italiano di Tecnologia, Via Fossato di Mortara 17-19, Ferrara, 44121 Italy
d) Azienda Ospedaliero Universitaria di Modena, Via del Pozzo 71, Modena, 41125 Italy

**Table S1:** aCSF composition

| NaCl | 127 mM |
| --- | --- |
| KCl | 1 mM |
| KH_2_PO_4_ | 1.2 mM |
| NaHCO_3_ | 26 mM |
| D-glucose | 10 mM |
| CaCl_2_ | 2.4 mM |
| MgCl_2_ | 1.3 mM |

a

b

cv

**Figure S1:** Electrochemical characterization of the gate electrode used as working electrode in a standard three-electrode cell. a) Cyclic voltammograms and b) Nyquist plot from EIS of the gate electrode at different functionalization steps. c) Nyquist plot showing the whole EIS for the gate electrode after passivation with OEG SAM. Bare Au electrode (black), Au functionalized with cys-protein G (red), after immobilization of the antibody (green), and after passivation with OEG SAM (blue).

a

b

c

**Figure S2:** a) Comparison between *ΔV_th_* (black circles) and *ΔV_ON_* (red circles). The dashed lines are the best fit with Langmuir model (equation 1). b) Relative variation of transconductance and c) variation of *σ* in function of [p-tau]. The continuous lines are the best fit with Langmuir model (equation 1). The error bars correspond to the standard deviation of 4 independent experiments.

**Table S2:** affinity constant values *K_a_* for the couple p-tau/antibody extracted for the different parameters (data from Figure 2 and Figure S2).

| **Data set** | **Ka** |
| --- | --- |
| ΔI/I_0_ @ V_GS_ = -0.4 V | (7.1±2) ⋅10^12^ |
| ΔI/I_0_ @ V_GS_ = -0.5 V | (3.6±1) ⋅10^12^ |
| ΔI/I_0_ @ V_GS_ = -0.6 V | (2.7±0.4) ⋅10^12^ |
| ΔV_th_ | (2.7±0.8) ⋅10^12^ |
| ΔV_ON_ | (1.2±0.2) ⋅10^12^ |
| Δg_m_/g_m0_ | (1.6 ±0.4) ⋅10^12^ |
| Δσ | (1.8 ±0.2) ⋅10^12^ |

**Figure S3:** Response of the EGOT sensor functionalized with specific anti-p-tau Ab (red) and non-specific Ab to the same concentration of p-tau protein in CSF (116 pg/mL). Data are reported as mean of 4 (red) and 3 (green) independent experiments (± standard deviation).

**Figure S4:** Relative variation of current response of the EGOT sensor functionalized with non-specific Ab as a function of concentration of p-tau in CSF. The error bars correspond to the standard deviation of 3 independent experiments.
